# Characterization and Molecular Determinants for β-Lactam Specificity of the Multidrug Efflux Pump AcrD from *Salmonella typhimurium*

**DOI:** 10.3390/antibiotics10121494

**Published:** 2021-12-06

**Authors:** Jenifer Cuesta Bernal, Jasmin El-Delik, Stephan Göttig, Klaas M. Pos

**Affiliations:** 1 Institute of Biochemistry, Goethe-University Frankfurt, Max-von-Laue-Str. 9, D-60438 Frankfurt am Main, Germany; jcuesta.bernal@gmail.com (J.C.B.); El-Delik@em.uni-frankfurt.de (J.E.-D.); 2 Institute of Medical Microbiology and Infection Control, Hospital of the Goethe University, Paul-Ehrlich-Straße 40, D-60596 Frankfurt am Main, Germany; Stephan.Goettig@kgu.de

**Keywords:** antibiotic resistance, efflux pump, RND

## Abstract

Gram-negative Tripartite Resistance Nodulation and cell Division (RND) superfamily efflux pumps confer various functions, including multidrug and bile salt resistance, quorum-sensing, virulence and can influence the rate of mutations on the chromosome. Multidrug RND efflux systems are often characterized by a wide substrate specificity. Similarly to many other RND efflux pump systems, AcrAD-TolC confers resistance toward SDS, novobiocin and deoxycholate. In contrast to the other pumps, however, it in addition confers resistance against aminoglycosides and dianionic β-lactams, such as sulbenicillin, aztreonam and carbenicillin. Here, we could show that AcrD from *Salmonella typhimurium* confers resistance toward several hitherto unreported AcrD substrates such as temocillin, dicloxacillin, cefazolin and fusidic acid. In order to address the molecular determinants of the *S. typhimurium* AcrD substrate specificity, we conducted substitution analyses in the putative access and deep binding pockets and in the TM1/TM2 groove region. The variants were tested in *E. coli* Δ*acrB*Δ*acrD* against β-lactams oxacillin, carbenicillin, aztreonam and temocillin. Deep binding pocket variants N136A, D276A and Y327A; access pocket variant R625A; and variants with substitutions in the groove region between TM1 and TM2 conferred a sensitive phenotype and might, therefore, be involved in anionic β-lactam export. In contrast, lower susceptibilities were observed for *E. coli* cells harbouring deep binding pocket variants T139A, D176A, S180A, F609A, T611A and F627A and the TM1/TM2 groove variant I337A. This study provides the first insights of side chains involved in drug binding and transport for AcrD from *S. typhimurium*.

## 1. Introduction

Antibiotic resistance has become a global public health concern due to the appearance of resistant strains, especially from pathogen Gram-negative bacteria, that acquired resistance determinants against many clinically used anti-infective agents. This phenomenon, known as Multidrug Resistance (MDR), can be caused by a simultaneous presence of multiple resistance mechanisms that are encoded on transferable plasmids or chromosomes [1]. Among these mechanisms, the increased active export of the drugs by multidrug efflux pumps can cause simultaneous resistance to several toxic compounds, representing a major challenge for new antibiotics development [2,3].

Gram-negative systems comprising inner membrane proteins from the Resistance Nodulation and cell Division (RND) superfamily play a major role in multidrug resistance because of their action of assembling into tripartite complexes that span the entire bacterial envelope and their ability to capture drugs from the periplasm and expelling these drugs towards the extracellular medium [3,4]. These RND-type tripartite systems play also other roles in biofilm formation, quorum-sensing, bile salt resistance and virulence, and their activity appears to be connected to the increase in mutations on the chromosome [5,6,7,8,9,10].

The importance of many (other) roles is evidenced by their wide distribution in all domains of life, and several RND pumps are genotypically encoded in the same organism in most cases [11]. At least five RND multidrug efflux pump genes have been identified in the *Escherichia coli* chromosome, ten have been identified in *Klebsiella pneumoniae,* two have been identified in *Campylobacter jejuni* and six have been identified in *Salmonella typhimurium* [12]. The potential for the deployment of these transporters in numerous bacterial species of clinical concern, such as the ESKAPE pathogens, has directed RND-type tripartite transporter research efforts toward these organisms in order to understand their structural and functional basis [2,4,13].

*Salmonella enterica* serovar Typhimurium (*S. typhimurium*) is a Gram-negative bacterium that causes disease in both humans and animals [14]. This food-borne pathogen accounts for the high incidence of *Salmonella* infections worldwide and emerging antibiotic resistance strains have been reported, representing a potentially serious public health problem [15]. *S. typhimurium* has at least nine functional drug efflux pumps belonging to different transporter (super)families. It has been reported that RND members, such as AcrB, AcrD, AcrF, MdsB and MdtBC, play a major role in the observed *Salmonella* resistance phenotype to a wide range of toxic compounds [14,16,17].

Several studies on the expression, regulation and transcriptome profiling of single and multiple deletion strains of RND efflux pump genes in *S. typhimurium* established that the main RND pump conferring antibiotic resistance is the AcrAB-TolC system, and its inactivation results in multidrug hypersusceptibility [14,16,17,18,19]. However, its loss (i.e., Δ*acrB*) is to a certain degree compensated by the increased expression of homologous pumps AcrD and AcrF [16].

AcrD from *S. typhimurium* (St_AcrD) assembles in a tripartite complex together with the Membrane Fusion Protein (MFP) AcrA (St_AcrA) and the Outer Membrane Factor (OMF) TolC (St_TolC), i.e., the same interaction partners employed by AcrB (St_AcrB), in order to export noxious compounds out of the cell, including SDS, novobiocin and deoxycholate [20], and also more hydrophilic compounds such as β-lactams (oxacillin, nafcillin, cloxacillin, carbenicillin, sulbenicillin and aztreonam) and aminoglycosides [20,21]. In contrast, these β-lactams and aminoglycosides are weak or non-substrates of the AcrB pumps in *E. coli* (Ec_AcrB) and *S. typhimurium* [20,22,23]. A molecular dynamics approach comparing Ec_AcrB and *E. coli* AcrD (Ec_AcrD) proximal (access) and distal (deep) binding pockets indicated that their volume and shape are rather similar [24]. However, Ec_AcrD comprises more polar and charged residues in the binding pockets compared to Ec_AcrB. By using chimeric AcrB/AcrD constructs and site-directed mutagenesis, it was shown that AcrB could confer increased resistance against aztreonam, sulbeniccilin and carbenicillin when three residues in the access pocket with an overall negative charge (1^−^) were exchanged for residues with an overall positive charge (2^+^). However, the molecular determinants for substrate binding to the RND transporter St_AcrD (or its closest homolog Ec_AcrD, with 94% identical residues) have not been experimentally characterized.

In this study, we used site-directed mutagenesis to target single side chains within the AcrD drug-binding access pocket, the deep binding pocket and the TM1/TM2 groove for substitution. Functional analysis in presence of monoanionic and dianionic β-lactams revealed several regions that are permissive towards substitution and that can cause hyperactive variants.

## 2. Results

### 2.1. Substrate Specificity of St_AcrD

AcrD from *S. typhimurium* has been functionally characterized and was shown to be dependent on AcrA and TolC for its activity [20]. Earlier studies had shown that the deletion of the *acrD* gene from the *S. typhimurium* chromosome resulted in susceptibilities towards aminoglycosides such as amikacin, gentamicin, neomycin, kanamycin and tobramycin and that Δ*acrD* cells accumulated higher levels of dihydrostreptomycin and gentamicin compared to the parental strain [23]. AcrAD-TolC has been shown to further confer resistance towards SDS, novobiocin and various β-lactams such as oxacillin, cloxacillin, nafcillin, carbenicillin, sulbenicillin and aztreonam.

Based on known RND multidrug efflux pump structures, we assume that AcrD is active as a trimer and that the protomers also might adopt different conformations. These different conformations might present substrate binding sites for drugs sequestered from the periplasm or the inner membrane. In order to obtain more detailed structural information on St_AcrD, we utilized a direct structural comparison with Ec_AcrB by using sequence alignments and homology model building, as both proteins share 66% identical residues (Table S1, Figure S1). The putative substrate binding pockets of St_AcrD were assigned according to the location and amino acid composition of its homologues Ec_AcrB and Ec_AcrD [24]. This study addresses residues from the Access Pocket (AP), Deep Binding Pocket (DBP) and fusidic acid binding site (TM1/TM2 region), and the latter specifically binds carboxylated drugs (fusidic acid and lipophilic β-lactams) in Ec_AcrB (Figures S1 and S2) [25].

In order to test St_AcrD functionality, drug susceptibility tests were performed with *E. coli* BW25113 (DE3) Δ*acrB*Δ*acrD*, since this strain does not express RND pump components AcrB and AcrD but still produces tripartite interaction partners AcrA and TolC. The expression plasmid p7XC3H-St_AcrD (pSt_AcrD) was employed to produce wildtype St_AcrD, and the cloning vector p7XC3H-Δ*ccdB* (pControl) was used as a negative control (see Supplementary Materials and Methods). The sequence identity of the tripartite complexes AcrAD-TolC of *E. coli* and *S. typhimurium* was high (Ec_AcrD vs. St_AcrD 94%, Ec_AcrA vs. St_AcrA 97% and Ec_TolC vs. St_TolC 90%). Hence, there is a fair assumption that St_AcrD assembles into a fully active tripartite efflux pump together with the components Ec_AcrA and Ec_TolC (encoded on the chromosome) and, therefore, complement the AcrB/AcrD deficient phenotype in *E. coli* [26].

**Figure 1:**
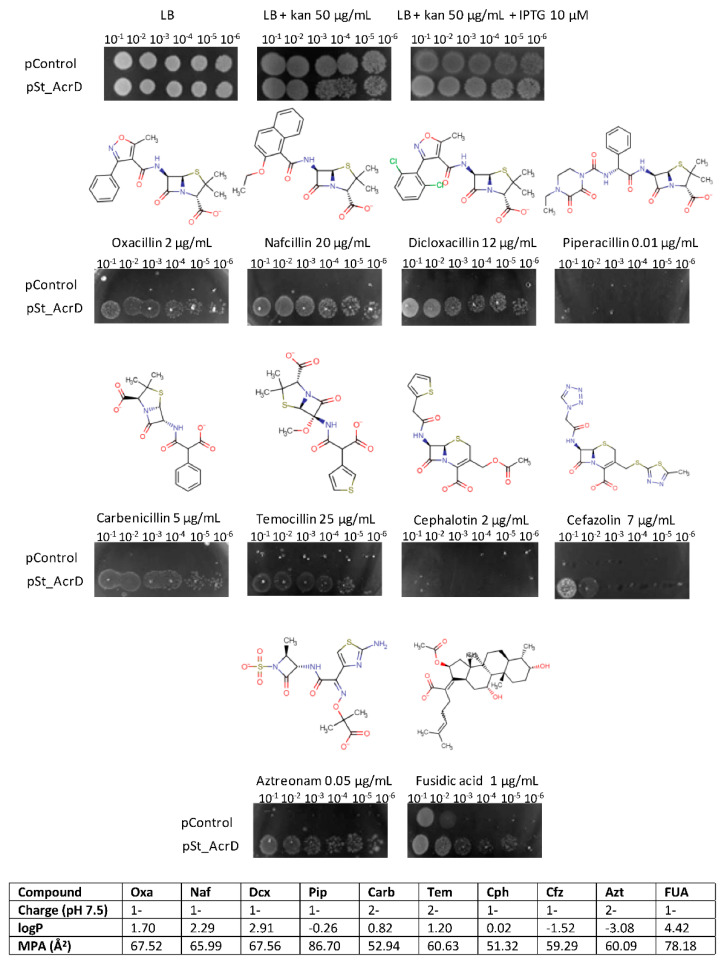
Determination of St_AcrD substrate specificity by agar plate dilution assay. Serial dilutions of normalized *E. coli* BW25113 (DE3) Δ*acrB*Δ*acrD* harboring the plasmids pControl (empty vector, negative control) or St_AcrD (pSt_AcrD) were spotted on LB agar plates supplemented with 50 μg/mL kanamycin, 10 μM IPTG and the indicated antibiotic and concentration. Physicochemical properties of tested substrates are indicated below (https://chemicalize.com/#/, accessed on 8 May 2018). Oxa: Oxacillin; Naf: Nafcillin; Dcx: Dicloxacillin; Pip: Piperacillin; Carb: Carbenicillin; Tem: Temocillin; Cph: Cephalotin; Cfz: Cefazolin; Azt: Aztreonam; FUA: Fusidic acid; logP: logarithm of the partition coefficient; MPA: minimal projection area.

Functionally active phenotypes of St_AcrD could be detected by the plate dilution assay, as cells that produced wildtype St_AcrD were able to grow at higher cell dilutions compared to the negative control (Δ*acrB*Δ*acrD*) in presence of anionic β-lactam (Figure 1). This included substrates such as oxacillin, carbenicillin, nafcillin and aztreonam, which were shown previously to be transported by the pump [20]. These observations also confirm the formation of a hybrid tripartite system St_AcrD-Ec_AcrA-Ec_TolC in *E. coli* [26]. Interestingly, we could identify several hitherto unreported substrates such as temocillin, dicloxacillin, cefazolin and fusidic acid. Furthermore, piperacillin does not appear to be a substrate for the AcrD pump (Figure 1).

The substrate specificity for St_AcrD as determined by the plate dilution method could be confirmed by Minimal Inhibitory Concentration (MIC) determinations [27] performed by the antibiotic gradient test for clinically relevant antibiotics (Table 1). In this assay, *E. coli* BW25113 (DE3) Δ*acrB*Δ*acrD* harboring pSt_AcrD conferred resistance against temocillin, oxacillin, aztreonam, cephalotin and cefazolin, exhibiting increases between 8-fold to 16-fold with respect to MIC values.

### 2.2. Site-Directed Mutagenesis of Substrate Binding Pocket Residues

In order to experimentally characterize substrate binding pockets in St_AcrD and to identify residues that are essential for β-lactam transport, single alanine mutants (Ala-mutants) were produced by site-directed mutagenesis. In total, 21 residues were selected and classified within three groups: deep binding pocket (DBP) residues (N136, T139, D176, Y178, S180, K274, D276, Y277, Y327, F609, S610, T611, S614 and F627), access pocket (AP) residues (R568, R625 and G672) and TM1/TM2 region residues (I27, I337, I338 and V341) (Figures S1 and S2).

The susceptible *E. coli* BW25113 (DE3) Δ*acrB*Δ*acrD* strain was transformed with plasmids encoding the indicated St_AcrD Ala-substitution variants and tested for activity in plate dilution assay (Figures S3–S5). Cells carrying Ala-variants grew to the same extent under non-selective conditions compared to cells harboring pSt_AcrD (WT) and pControl (Figure S3). The plate dilution assay is a preferred method for the analysis of subtle activity changes due to single-site substitution, as it is a direct visualization of the bacterial growth rate. In the experiments shown in Figure S4, the LB agar plates in addition contained kanamycin as selective antibiotic for the selection marker on the pSt_AcrD plasmid, whereas the LB agar plates in the experiments shown in Figure S5 did not contain kanamycin.

**Figure 2:**
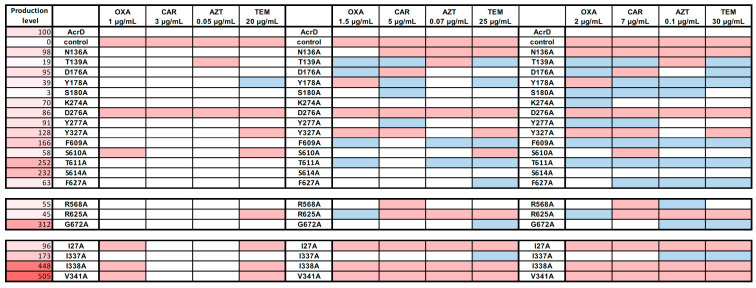
Relative susceptibilities toward β-lactams of *E. coli* Δ*acrB*Δ*acrD* complemented by AcrD variants. Oxacillin (OXA), carbenicillin (CAR), aztreonam (AZT) and temocillin (TEM) were used at the concentrations indicated. Based on visual inspection of the number of spots on the plates for each variant shown in Figures S4 and S5, each variant was indicated as compromised (red shade), hyperactive (blue shade) or showed the same phenotype compared to cells harbouring wildtype AcrD. Relative production levels of each variant are indicated on the left based on the values determined via Western Blot analysis (Figure S7). The wildtype production level was set to 100, the control was set to 0.

All variants were tested in the presence of the four β-lactams, oxacillin, carbenicillin, temocillin and aztreonam, in order to identify whether mutants could complement the *E. coli* susceptible phenotype, as was shown for wildtype St_AcrD. Either two similar or two opposite phenotypes were observed compared to the wildtype: Hyperactive mutants that grew on higher antibiotic concentrations or exhibited more copious growth as observed by denser and more intense spots on the LB-agar-plate. On the other hand, compromised mutants are those that were not able to grow to the same extent as cells producing wildtype St_AcrD (Figure 2, Figures S4 and S5).

#### 2.2.1. Deep Binding Pocket Variants

Depending on the antibiotic stress applied, approximately half of the DBP variants exhibited similar growth compared to cells with wildtype St_AcrD; however, hyperactive (T139A, D176A, Y178A, S180A, F609A, T611A and F627A) and sensitive mutants (N136A, D276A and Y327A) were identified (Figure 2, Figures S4 and S5). Most of these observations were consistent for the substrates tested (either sensitive or hyperactive), which could indicate that monoanionic and dianionic β-lactams might interact with the same residues located in the DBP. In contrast, T139A shows hyperactivity towards oxacillin, carbenicillin and temocillin but was not able to transport aztreonam. This suggests that the hydroxyl group of T139 is essential for aztreonam translocation along the substrate pathway. Likewise, Y178A confers a hyperactive phenotype, except toward oxacillin, where growth is impaired (Figure 2). Moreover, F609A conferred hyperactivity, whereas the homologous Ec_AcrB substitution variant F610A was previously shown to confer most considerable sensitivity towards all tested AcrB substrates. In fact, for AcrB, the F610A substitution had the strongest effect on the MIC values of all DBP substitutions tested [28]. One interpretation for the latter variant might be that the lack of the phenyl group facilitates transport of dianonic substrates in this St_AcrD variant, permitting accommodation of the more hydrated hydrophilic compounds in DBP [24]. On the other hand, a decrease in polar environment in the DBP (T139A, D176A, S180A and T611A) also resulted in hyperactive phenotypes. These residues are distributed in the core of the DBP PN2 and PC1 subdomains of St_AcrD, and their Ec_AcrB counterparts interact directly with doxorubicin and minocycline in the DBP, as shown in co-crystal structures [29] (Figure S6). Most distal in the DBP, D276 seems to be involved either in direct interaction with β-lactams or their transport through the exit tunnel, as the D276A substitution resulted in the most pronounced sensitivity towards all four β-lactams. Notably, an *E. coli* Δ*acrB* strain complemented with an Ec_AcrB D276C variant showed wildtype-like MIC values for erythromycin and novobiocin [30]. Residues in the other sensitive variants, i.e., N136A, Y327A and the aztreonam sensitive T139A variant, were all proximally located in the PN2 subdomain and close to the AP, where substrate recognition of anionic β-lactams has been suggested to take place [21].

Protein production of St_AcrD WT and Ala-variants in *E. coli* was detected by Western blot analysis (Figure 2 and Figure S7). The activity of the tripartite efflux system will not only depend on the concentration of the RND component but also on the available periplasmic concentration of interaction partners AcrA and TolC. Sensitive mutants N136A, D276A and Y327A were produced at similar levels compared to wildtype St_AcrD; hence, a decrease in activity is most likely directly related to the substitution. On the other hand, the increased activity observed for the mutants F609A and T611A might be partially explained by the overproduction of these variants compared to St_AcrD WT. However, there appears to be no clear correlation between activity and production levels as another variant, S614A, was overproduced to the same extent as T611A and showed mostly wildtype-like resistance phenotypes (Figure 2, Figures S4 and S5). Furthermore, despite the low level protein production of T139A, Y178A and even more surprisingly S180A, higher wildtype resistances could be observed under most conditions (Figure 2). This indicates that the concentration of St_AcrD in cells was not limiting for exhibiting wildtype phenotypes, whereas it cannot be excluded that overproduction might still result in higher resistance phenotypes.

#### 2.2.2. Access Pocket Variants

The AP variant R625A conferred decreased activity against dianionic β-lactams, such as carbenicillin, temocillin and aztreonam, but not the monoanionic oxacillin (Figure 2, Figures S4 and S5). This is in line with the observation that R625 is important for the recognition of negatively charged β-lactams in Ec_AcrD [21]. Despite the fact that Western blot analysis shows reduced expression of this mutant (Figure 2, Figure S7), R568A conferred a clear hyperactive phenotype towards aztreonam (at 0.1 mg mL^−1^) and moderate hyperactivity towards temocillin, whereas a somewhat decreased resistance towards carbenicillin was observed (Figure 2, Figures S4 and S5). The G672A variant conferred a hyperactive phenotype towards temocillin and aztreonam and showed higher production levels compared to the wildtype (Figure 2 and Figure S7).

#### 2.2.3. Substitution Variants in the TM1/TM2 Groove

The I27A variant exhibited the most sensitive phenotype for all tested antibiotics (Figure 2 and Figures S4 and S5). On the other hand, the St_AcrD I337A variant appears to confer a hyperactive phenotype (for aztreonam and temocillin). In contrast, the counterpart I337A substitution in Ec_AcrB resulted in a marked increased sensitivity towards β-lactam antibiotics, including oxacillin [25,31]. The overproduction of St_AcrD I337A (1.7-fold compared to St_AcrD WT, Figure S7) may possibly obscure its potential reduced ability to transport β-lactams. On the other hand, variants I338A and V341A, both conferring substantially reduced resistance towards all tested β-lactams, showed far more extensive overproduction in *E. coli* (4.5-fold and 5-fold, respectively). Thus, a clear correlation between overproduction and mutant phenotype was not warranted. Another consideration might be that substitutions can cause improper protein folding and insertion in the membrane, causing a large signal via Western blot analysis, which does not necessarily indicate proper folding. For wildtype St_AcrD and five other RND efflux pumps, correct folding was analyzed by using RND-GFP fusions (Supplementary Materials and Methods), where proper folding is indicated by the GFP fluorescent signal [32] (Figure S8). Subsequent in-gel fluorescence in combination with Western Blot analysis indicated that the ratio of well-folded, fluorescent St_AcrD and misfolded proteins is approximately 1:1 (Figure S9). Moreover, AcrD could be purified from the AcrD-overproducing cells and results in a clear monodisperse peak indicating no aggregation in a solubilized state even after storage at 4 °C or 17 °C for one week (Figure S10). Comparison with purified AcrB indicates that AcrD is present as a monomer in detergent solution (Figure S11). Future studies are necessary in order to confirm the ratio between well-folded and misfolded proteins inside the cell membrane of each of the St_AcrD variants.

In summary, St_AcrD with Ala-substituted residues N136, D276 and Y327 in the deep binding pocket confers an overall sensitive phenotype towards the tested antibiotics, whereas T139A and Y178A confer a selective and higher resistance phenotype, as growth in aztreonam (T139A) or oxacillin (Y178A) was impaired. F609A conferred an overall higher resistance phenotype towards all tested substrates. This observation might possibly be in line with the reduction in hydrophobic binding pocket environment resulting in the enhancement of more hydrophilic substrate transport. Ala-substitutions made in the TM1/TM2 groove, proposed to be the initial binding site for carboxylated drugs in AcrB [25,31], conferred higher susceptibility to all four β-lactams tested (Figure 2, Figure S5). As an exception, I337A was shown to confer either wildtype-like or reduced susceptibility towards these substrates.

## 3. Discussion

Recently, a major emphasis was placed on structure elucidation of different RND proteins [4,13]. Nevertheless, structure–function relationships are far from clear and are in need of analysis via mutational analysis, biochemical/biophysical substrate binding, transport studies and molecular dynamic simulations. In particular, the latter technique not only requires high-resolution structures but is also reliant on additional experimental data for a supply of calculation setups with restraints derived from experiments. The results obtained from the simulation data then can be fed back into the experimental design [33].

Here, we addressed the substrate specificity for AcrD from *Salmonella typhimurium* and conducted functional analysis of AcrD variants with Ala-substituted binding pocket residues. For wildtype AcrD, we could identify temocillin, dicloxacillin, cefazolin and fusidic acid as substrates for the pump, whereas piperacillin is not a substrate (Figure 1, Table 1). The results for wildtype AcrD between plate dilution and MIC (antibiotic gradient) assays were congruent, except for cephalotin where resistance was not observed in the plate dilution assay, whereas the antibiotic gradient test showed a clear increase in MIC in AcrAD-TolC producing cells (Figure 1, Table 1).

Amongst the antimicrobials for which AcrD conferred less susceptibility, lipophilicity showed a wide distribution on the basis of the logarithm of the partition coefficient, logP [34]. St_AcrD can not only transport very hydrophilic β-lactams such as aztreonam but also the very hydrophobic antimicrobial fusidic acid. Both substrates have the presence of a carboxylic acid moiety as a common feature [25]. As an exception, piperacillin was not transported by St_AcrD despite its low logP. Piperacillin exhibits the largest minimal projection area (MPA) among the tested substrates such that it can be assumed that this characteristic might hamper its transport [35]. However, the latter property is not an impediment for the substrate to be exported by Ec_AcrB (through TM1/TM2 region). Furthermore, the volume of the substrate binding pockets of both proteins is expected to be similar and large enough to accommodate the largest reported substrates [24]. It is, hence, probable that additional non-conserved residues in the substrate translocation pathway of St_AcrD might play a substantial role. An additional consideration is the protonation state of the transported compounds at neutral pH. In contrast to AcrB, which preferentially transports monoanionic species of β-lactams, St_AcrD is able to transport monoanionic and dianionic β-lactams. Thus, it might be possible that the substrate pathway of both charged species towards the binding pockets might differ. This has been recently shown for AcrB comprising at least four channels toward the drug binding sites. These channels displayed preferences for substrates on the basis of their physicochemical properties. [21,31].

In order to characterize the role of residues inside substrate binding pockets, we conducted substitution analysis of St_AcrD. Residues that are part of the putative translocation pathway and for the putative substrate binding sites were identified (Figure S2) and substituted by Ala for a systematic characterization. Targeted residues were principally polar or charged, as it was expected that they are involved in electrostatic interactions with the negatively charged substrates. Additionally, several more hydrophobic amino acids were also investigated, including residues in the transmembrane domain, as β-lactams are partially immersed in the outer leaflet of inner membrane of *E. coli* [34].

**Figure 3:**
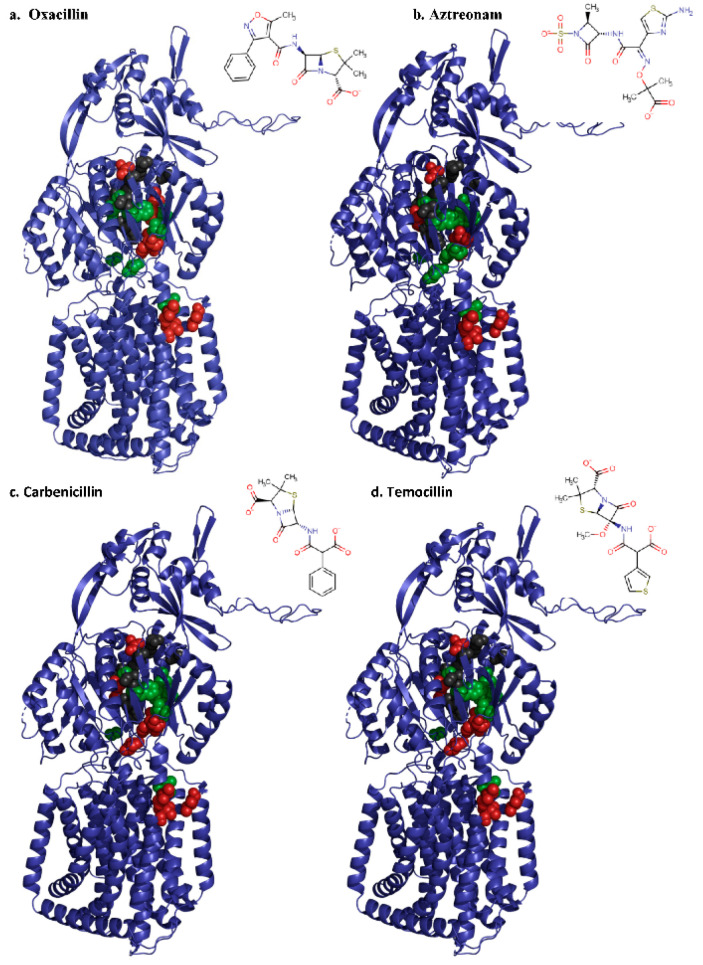
Putative substrate translocation pathway of the RND transporter St_AcrD. Homology model of St_AcrD localizing selected residues in DBP, AP and TM1 and TM2 regions involved in β-lactam transport. Ala-variants of selected residues exhibited hyperactive (green spheres), defective (red spheres) or similar phenotype (grey spheres) compared with wildtype St_AcrD in the presence of (**a**) oxacillin, (**b**) aztreonam, (**c**) carbenicillin and (**d**) temocillin. Figures were prepared with Pymol (https://pymol.org/, accessed on 9 May 2018).

We observed that substitutions did not only reduce resistance (conferred higher susceptibility) but also indicated hyperactivity for some of the substituted variants (Figure 2). Substrate specificity determinants appear not only to be limited to residues located in the AP as previously reported [21,31] but also include residues from the DBP and the TM1/TM2 region (Figure 3). One of the most surprising observations was related to the variants F609A and I337A, which exhibited a hyperactive phenotype, in strong contrast with its *E. coli* AcrB variant counterparts (F610A and I337A), which were shown to confer increased susceptibility [25,28]. For F609A, the hyperactivity might be in line with a reduction in the hydrophobic binding pocket environment resulting in the enhancement of the transport of hydrophilic substrates. For I337A, which shows a moderate overproduction compared to wildtype (Figure 2), the difference in phenotype between AcrB and AcrD is less straightforward to interpret. These results also emphasize the importance of studying single substitution variants of other RND pumps and directly comparing their effects (rather than extrapolate from one homolog RND pump to another), as AcrB and AcrD same-site substitution variants display different effects on susceptibility. This study emphasizes that substitutions of homologue residues can even result in opposite observations, as is shown for the St_AcrD F609A and I337A variants.

As expected, some substitution variants of originally charged polar residues such as N136A and D276A in DBP as well as R625A in the AP exhibited a sensitive phenotype and might, therefore, be involved in anionic β-lactam export. Moreover, the I27A mutants located in TM1/TM2 and Y327A in the DBP (except for aztreonam) were unable to complement the *E. coli*-susceptible phenotype in the presence of tested compounds (Figure 2 and Figure 3). This is opposed to previous suggestions that aromatic residues in DBP would not influence specificity towards anionic β-lactams [21]. Nevertheless, the effect of the substitution can also be due to secondary structural effects since the geometry of adjacent residues might be influenced by altered physicochemical properties. We also observed a high number of single-site variants displaying a hyperactive phenotype, which in some cases could be associated with increased protein production in the cell (Figure 2). The effect of overproduction, i.e., higher number of RND molecules might be directly correlated to the observed activity. Nevertheless, in cases such as T139A, S180A and Y178A, production levels were much lower compared to WT expression, yet these variants confer a considerable reduced susceptibility towards some of the antibiotics tested. Future studies should establish the cause of variable production of the variant proteins in the cell and whether the observed results are correlated with correct insertion in the inner membrane (i.e., as a well-folded and active transporter).

Although this study focused on β-lactam specificity, the presented system and constructed Ala-variants are potentially available for investigating additional substrates and inhibitors. The identification of key residues and interactions involved in substrate recognition along the translocation pathway, including the switch loop [29,36] and the most proximal residues in the AP, would help to understand the determinants of polyspecificity of RND multidrug transporters and the mechanisms by which substrate uptake occurs.

## 4. Materials and Methods

### 4.1. Site-Directed Mutagenesis

Site-directed mutagenesis of the *St_acrD* gene to produce Ala-variants constructs was performed by inverse PCR with 2 ng of p7XC3H-St_AcrD and forward/reverse primers (0.5 µM each) (Table S2) Phusion Flash High Fidelity Mix (containing modified Phusion Hot Start II Polymerase, Thermo Fisher, Dreieich, Germany) using an initial denaturation step for 2 min at 98 °C, followed by 30 cycles of denaturation/annealing/extension at 98 °C/65 °C→50 °C (touchdown)/72 °C for 20/30/130 s and a final extension step for 10 min at 72 °C. The resulting PCR product was digested with 10 U *DpnI* in 1× Fast Digest buffer for 2 h at 37 °C, followed by enzyme inactivation at 80 °C for 20 min. The PCR product was purified with the DNA Clean and Concentrator Kit (Zymogen, Freiburg, Germany) and employed in a simultaneous phosphorylation and ligation reaction: 10 µL of PCR product, 1 U T4 ligase, 1× T4 ligase buffer and 10 U T4 polynucleotide kinase. The reaction was incubated o/n at room temperature and later employed to transform *E. coli* MC1061 chemically competent cells. Single colonies were selected for o/n cultures and plasmid isolation with QIAprep Spin Miniprep Kit (QIAGEN, Hilden, Germany).

### 4.2. Antimicrobial Susceptibility Testing: Plate Dilution Assay and MIC Determination

Freshly transformed *E. coli* BW25113 (DE3) Δ*acrB*Δ*acrD* cells with expression constructs p7XC3H to produce St_AcrD or its Ala-variants were used to inoculate 2 mL of LB supplemented with 50 µg/mL kanamycin. Cultures were incubated o/n at 37 °C and 180 rpm. On the next morning, cultures were normalized to OD_600_ = 1 and serially diluted from 10^−1^ to 10^−6^ in 96-well plates and kept at 4 °C until use. LB-agar plates containing the tested antibiotics were prepared by dissolving the antibiotics (1, 1.5 and 2 µg/mL oxacillin; 3, 5 and 7 µg/mL carbenicillin; 20, 25 and 30 µg/mL temocillin; and 0.05, 0.07 and 0.1 µg/mL aztreonam) and 10 µM IPTG solutions into hand-warmed LB-agar supplemented with 50 µg/mL kanamycin. Medium was poured in single well Omnitray Plate (Thermo Fisher, Dreieich, Germany). A volume of 4 µL of the cell suspension dilutions previously prepared was spotted on LB-agar-antibiotic plates. Once the drops were dry, plates were incubated 16 h at 37 °C. On the next day, the results were evaluated visually, and images were taken with Image Quant LAS4000 Imager (GE Healthcare, Solingen, Germany). The determination of the minimum inhibitory concentration (MIC) was performed by employing antibiotic gradient strips (Liofilchem, Roseto degli Abruzzi, Italy). Mueller–Hinton plates supplemented with 50 µg/mL kanamycin and 10 µM IPTG were inoculated with a fresh bacterial suspension equivalent to a 0.5 McFarland standard. After application of antibiotic gradient strips, plates were incubated for 20 h under aerobic conditions at 36 ± 1 °C, and MIC was determined according to the manufacturer’s recommendation (at crossing point of bacterial lawn and strip).

## Figures and Tables

**Table 1:** MIC determination of *E. coli* BW25113 (DE3) Δ*acrB*Δ*acrD* harboring pSt_AcrD or pControl. MIC determinations were performed by employing antibiotic gradient strips on Mueller–Hinton agar plates, supplemented with 50 µg/mL kanamycin and 10 µM IPTG. Experiments were performed in duplicate.

	MIC (µg/mL)	
Antibiotic	pControl	pSt_AcrD
Ampicillin	0.75	1
Temocillin	0.75	6
Piperacillin	0.047	0.064
Oxacillin	0.125	2
Penicillin	8	8
Mecillinam	0.023	0.032
Aztreonam	0.032	0.25
Cefotaxime	<0.016	<0.016
Cefoxitin	0.5	1
Cefepime	≤0.016	0.016
Ceftriaxone	0.023	0.016
Cefuroxime	0.125	0.125
Ceftaroline	≤0.016	0.016
Ceftazidime	0.047	0.047
Cefazolin	2	16
Cephalotin	2	12
Cefpodoxime	0.064	0.094
Imipenem	0.125	0.25
Doripenem	0.032	0.064
Meropenem	0.023	0.023
Tetracycline	0.25	0.25
Fusidic acid	2	4
Erythromycin	0.75	1.5
Chloramphenicol	0.5	0.38

## Data Availability

The data presented in this study are available upon request from the corresponding author.

## Supplementary Materials

The following are available online at https://www.mdpi.com/article/10.3390/antibiotics10121494/s1, Supplementary Materials and Methods, Figure S1. Homology model of St_AcrD, Figure S2. Putative substrate translocation pathway in St_AcrD and localization of the selected side chains for substitution, Figure S3. Plate dilution assay with *E. coli* BW25113 (DE3) Δ*acrB*Δ*acrD* harboring St_AcrD WT or the indicated Ala-substitution variants, Figure S4. Resistance profiles against β-lactams for *E. coli* BW25113 (DE3) Δ*acrB*Δ*acrD* harboring St_AcrD Ala-substitutions in the putative DBP, AP and in the TM1/TM2 region, Figure S5. Resistance profiles against β-lactams for *E. coli* BW25113 (DE3) Δ*acrB*Δ*acrD* harboring St_AcrD Ala-substitutions in the putative DBP (a. and b.) and AP and in TM1/TM2 region (c.), Figure S6. Comparison of DBP of Ec_AcrB (PDB: 4DX7) and St_AcrD (homology model), Figure S7. Production of St_AcrD WT and Ala-variants in *E. coli* BW25113 (DE3) Δ*acrB*Δ*acrD,* Figure S8. *E. coli* strains and inducer concentration screening to produce RND-GFP fusion proteins, Figure S9. *In gel fluorescence* and anti-His Western blot detection of RND-GFP fusion proteins produced in *E. coli*, Figure S10. Purification of St_AcrD, Figure S11. Oligomeric state of St_AcrD; Table S1: Comparison of identities and similarities of RND multidrug transporters of *E. coli*, *C. jejuni* and *S. typhimurium*, Table S2: Primers used for FX-cloning and site-directed mutagenesis, Table S3. Multidrug RND transporters from *Campylobacter jejuni* and *Salmonella typhimurium*, Table S4. Cloning and expression constructs of RND transporters produced by FX-cloning, Table S5. Summary of optimized conditions to produce RND-GFP fusion proteins in *E. coli*.
