# Supplementary material for: Characterization and Molecular Determinants for β-Lactam Specificity of the Multidrug Efflux Pump AcrD from Salmonella typhimurium
Source: Antibiotics (Basel). 2021 Dec 6;10(12):1494. doi: 10.3390/antibiotics10121494 (PMC8699017; doi:10.3390/antibiotics10121494)
Supplement: Supplementary file 1 [file antibiotics-10-01494-s001.zip › Supplementary Material and Methods_2021_11_11.pdf]

Characterization and molecular determinants for β-lactam specificity of the multidrug efflux pump AcrD from *Salmonella typhimurium*

Jenifer Cuesta Bernal[1], Jasmin El-Delik[1], Stephan Göttig[2], Klaas M. Pos[1*]

[1] Institute of Biochemistry, Goethe-University Frankfurt, Max-von-Laue-Str. 9, D-60438 Frankfurt am Main, Germany.

[2] Institute of Medical Microbiology and Infection Control, Hospital of the Goethe University, Paul-Ehrlich-Straße 40, D-60596 Frankfurt am Main, Germany.

*Corresponding author

Supplementary Materials and Methods

In silico selection of RND multidrug efflux pump for pre-crystallization screening

A total of eight genes encoding for secondary transporters classified within the RND HAE-1 family were annotated in the genomes *of Campylobacter jejuni* (*C. jejuni*) and *Salmonella enterica* serovar Typhimurium *(S. typhimurium)* (http://www.tcdb.org/, http://www.membranetransport.org/ and http://www.xbase.ac.uk/genome). Nucleotide and primary sequences of corresponding proteins were retrieved from NCBI https://www.ncbi.nlm.nih.gov/ (Table S3).

PCR

RND target genes were amplified from *Campylobacter jejuni* or *Salmonella enterica* serovar Typhimurium LT2 (ATCC) genomic DNA (100 ng), using Buffer HF (ThermoFischer) with 200 µM dNTPs, 0.5 µM forward and reverse primers (Table S2) and 1 U of Phusion DNA Polymerase. After an initial denaturation step of 98 C /2 min, 35 cycles of denaturation/annealing/extension (98/61/72°C – 20/30/100 s)) was employed, concluded by a final extension step at 72°C for 10 min.

FX-cloning and sub-cloning of RND transporters

Initial cloning of target genes (Table S3, Table S4) was performed by FX-cloning into the cloning vector pINITcat. Digestion and ligation of PCR product insert (i) and vector (v) were done consecutively in the same tube using the following reaction conditions [1]: 50 ng of vector were mixed with 1:5 (v:i) molar ratio of insert (PCR product), 1x Tango buffer (Thermo Fisher) and 2.5 U *LguI* (SapI). Digestion was carried out for 2 h at 37°C, followed by inactivation of the enzyme at 65°C for 20 min. For ligation reaction, 10 mM ATP and 1 U T4 ligase were added to the mixture and incubated at 22°C for 1 h. After enzyme inactivation (20 min at 65°C), 10 µL of the ligation mixture were employed to transform *E. coli* MC1061 chemically competent cells. Single colonies were selected for o/n cultures and plasmid isolation was done with QIAprep Spin Miniprep Kit (QIAGEN). For FX-subcloning of RND target genes into expression vectors, the same protocol was applied; in this case pINITcat-RND constructs were employed as inserts in a molar ratio 1:4 (vector:insert).

DNA quantification and sequencing

Linear and plasmid DNA was quantified by UV-absorbance measurements at 260 nm using Nanodrop (Peqlab Biotechnologie GmbH). Cloning and expression constructs were sequenced by Eurofins Genomics and GATC Biotech companies.

Heterologous production screening of RND transporters in *E. coli*

One colony of freshly transformed *E. coli* cells (BL21 (DE3), C43 (DE3) Δ*acrAB*, C41 (DE3) Δ*acrAB* or MC1061) with membrane protein-green fluorescent protein (MP-GFP) expression constructs (Table S4, Table S5), was used to inoculate 1 mL LB supplemented with appropriate antibiotic, in 2 mL Eppendorf tube and incubated o/n at 37°C, 900 rpm in a thermomixer. Next day, o/n cultures were diluted 100x in 700 µL fresh LB media in 96-deep well plate format. The plate was covered with gas permeable membrane (Adhesive film for cultures plates porous, breathable rayon film non-sterile, VWR) and incubated at 37°C in a plate shaker incubator at 900 rpm. OD_{600} was monitored and when reached 0.5-0.6, cells were induced with IPTG (0.2-1.0 mM) or L-arabinose (0.0002-0.2% (w/v)). Cells were allowed to grow for 4 h at 25°C, in a plate shaker incubator at 900 rpm. After that, the 96-deep well plate was centrifuged at 4000 rpm (3046 g, rotor Microplate MP 3300) for 20 min to collect the cell pellets. Cells were resuspended in 200 µL PBS buffer (137 mM NaCl, 2.7 mM KCl, 10 mM Na_2HPO_4, 2 mM KH_2PO_4, pH 7.4) and divided in two samples: 1. 100 µL of cells from each well were transferred to a 96-black well plate (Brand plates PureGrade -S 781668) and whole cell fluorescence was measured in a TECAN Infinite M200 reader (Excitation wavelength 485 nm, emission wavelength 535 nm, gain 100). 2. The remaining 100 µL of cells from each well were transferred to a 96-transparent well plate (Brand plates 781602) and OD_{600} was measured in a TECAN Infinite M200 reader. Fluorescence was normalized to the OD_{600} value [42]. Once a suitable *E. coli* strain and inducer concentration were found for a specific RND protein production, the conditions were replicated for post-induction time and temperature screening (Table S5). This time, o/n cultures of freshly transformed cells were diluted to 10 mL fresh LB in 50 mL falcon tubes and incubated at 37°C, 180 rpm. OD_{600} was monitored and when reached 0.5-0.6, cells were induced with appropriate inducer (IPTG or L-arabinose) at the indicated concentrations. Cell growth was continued at 25°C and 37°C, normalized cell samples (1 mL of cells of OD_{600}=1) were taken after 2, 4, 16 h, and centrifuged at 13000 rpm (12664 g, rotor Heraeus Sepatech 3743) for 5 min. Cells were resuspended in 200 µL PBS buffer; whole cell fluorescence and OD_{600} were measured as described above.

2.3 Production and purification of St_AcrD

E. coli C43 (DE3) Δ*acrAB* cells were transformed with p7XC3H-acrD construct and employed to inoculate 100 mL TB medium supplemented with 50 µg/mL kanamycin. Culture was incubated at 37°C, 180 rpm until OD_{600} reached 1-1.3 and then diluted 100x in fresh medium. Cell growth was continued at 37°C and 150 rpm, monitoring OD_{600} until 0.8-1.2 was obtained. At this point, cultures were cooled down at 4°C during 30 min and induced with 0.5 mM IPTG.

Cells were allowed to grow o/n at 25°C, 150 rpm. Next morning, cells were harvested by centrifugation 5500 rpm (6425 g, rotor FiberLite F8-6x1000y), for 15 min, 4°C and resuspended in lysis buffer (20 mM Tris-Cl, pH 7.5, 200 mM NaCl,1 mM $MgSO_4$, 0.5 mg/mL lysozyme, 100 μM Pefabloc SC and trace amounts of DNase I). Cells were lysed by passing 2 times through a Pressure Cell Homogenizer EP (Stansted) at 14-29 KPsi. Cell lysate was cleared by centrifugation (11730 rpm (20000 g, rotor FiberLite F14-6x250y), 30 min, 4°C) removing cell debris. To isolate the membrane fraction, the supernatant was centrifuged at 185000 g for 1 h, 4°C (40000 rpm, rotor Beckman Ti45). The membrane pellet was resuspended in membrane resuspension buffer (20 mM Tris-Cl, pH 7.5, 200 mM NaCl, 10 mM imidazole) employing a homogenizer (Potter-Elvehjem tube and PTFE pestle shearing). Membranes were aliquoted, frozen with liquid nitrogen and stored at -80°C. Thawed membrane was solubilized with 2% (w/v) DDM with mild agitation for 1 h at 4°C. Unsolubilized material was removed by centrifugation at 160000 g, 4°C for 45 min (40000 rpm, rotor Beckman Ti60). The supernatant was filtered through a 0.22 μm filter (Cellulose acetate, VWR), and loaded into His TRAP HP column (GE Healthcare) connected to a peristaltic pump. Flow-through was collected and column was washed with 20 mM Tris-Cl, pH 7.5, 0.04% (w/v) DDM buffers with increasing concentrations of imidazole (30, 60, 80 mM). Protein was eluted in 20 mM Tris-Cl pH, 7.5, 0.04% (w/v) DDM and 300 mM imidazole buffer and concentrated (Amicon Ultra 4, 100 kDa cutoff, Merck Millipore) before injection into Superose 6 15/300 increase column (GE Healthcare) coupled to and Äkta system and previously equilibrated in GF buffer (20 mM Tris-Cl, pH 7.5, 10 mM NaCl and 0.04% (w/v) DDM).

Homology model building

The tertiary structure of St_AcrD was predicted by homology modeling employing Phyre2 server[2]. As templates, high resolution L and T protomers of Ec_AcrB structure solved at 1.9 Å (PDB: 4DX5) were selected. The program covered more than 99% of the sequences. The quality of the homology models of St_AcrD in the L and T conformations, was validated by the servers PSVS [3] and SAVES v5.0 (https://saves.mbi.ucla.edu).

1. Geertsma, E.R.; Dutzler, R. A versatile and efficient high-throughput cloning tool for structural biology. *Biochemistry* **2011**, *50*, 3272-3278.
2. Kelley, L.A.; Mezulis, S.; Yates, C.M.; Wass, M.N.; Sternberg, M.J. The Phyre2 web portal for protein modeling, prediction and analysis. *Nat Protoc* **2015**, *10*, 845-858.
3. Bhattacharya, A.; Tejero, R.; Montelione, G.T. Evaluating protein structures determined by structural genomics consortia. *Proteins* **2007**, *66*, 778-795.
