# Supplementary material for: Characterization and Molecular Determinants for β-Lactam Specificity of the Multidrug Efflux Pump AcrD from Salmonella typhimurium
Source: Antibiotics (Basel). 2021 Dec 6;10(12):1494. doi: 10.3390/antibiotics10121494 (PMC8699017; doi:10.3390/antibiotics10121494)
Supplement: Supplementary file 1 [file antibiotics-10-01494-s001.zip › Supplementary_Figures_2021_11_12.pdf]

Characterization and molecular determinants for β-lactam specificity of the multidrug efflux pump AcrD from *Salmonella typhimurium*

Jenifer Cuesta Bernal[1], Jasmin El-Delik[1], Stephan Göttig[2], Klaas M. Pos[1*]

[1] Institute of Biochemistry, Goethe-University Frankfurt, Max-von-Laue-Str. 9, D-60438 Frankfurt am Main, Germany.

[2] Institute of Medical Microbiology and Infection Control, Hospital of the Goethe University, Paul-Ehrlich-Straße 40, D-60596 Frankfurt am Main, Germany.

*Corresponding author

Supplementary Figures

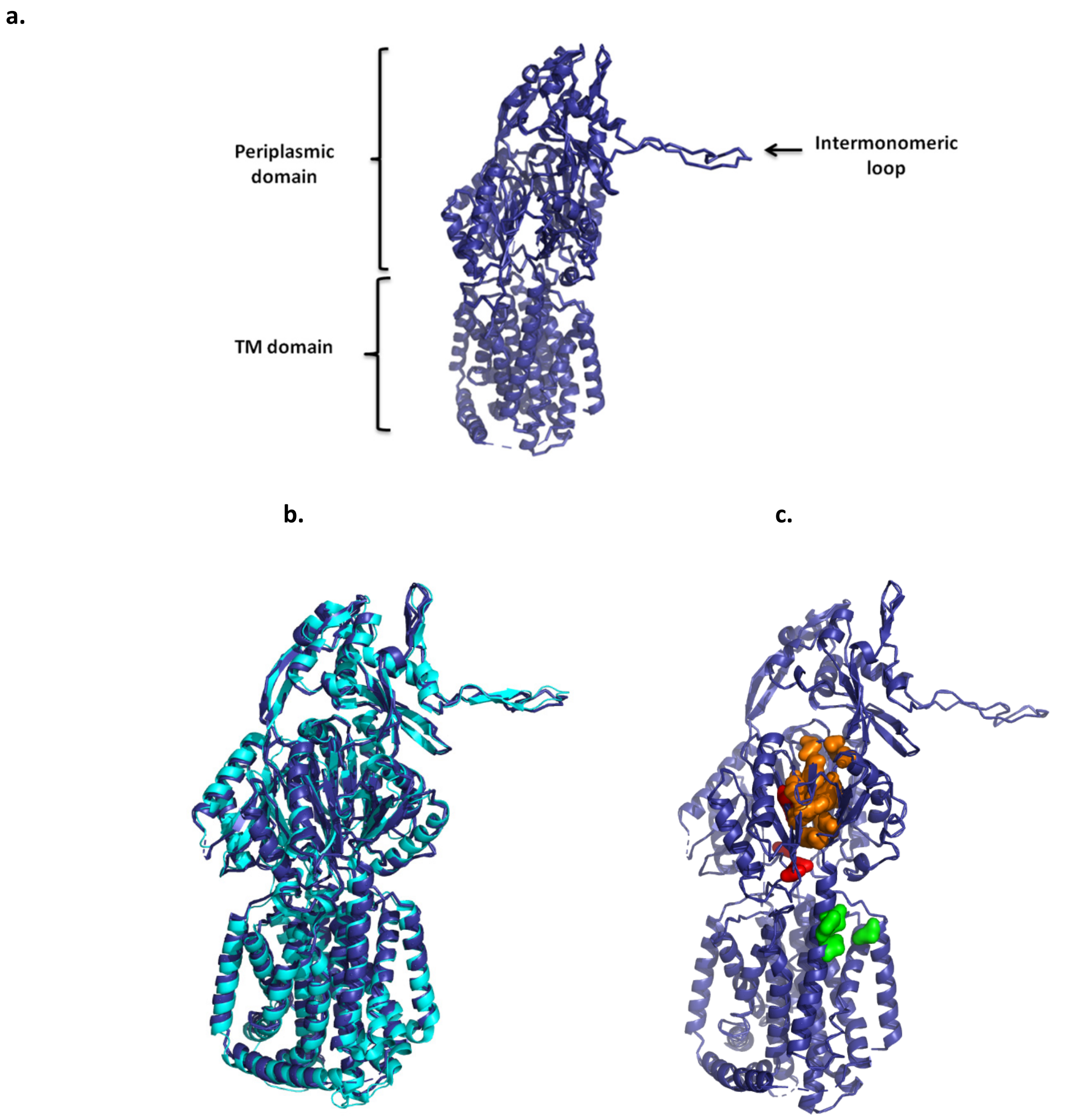

Figure S1. Homology model of St_AcrD. a. *In silico* model of St_AcrD was produced by Phyre2 server (http://www.sbg.bio.ic.ac.uk/phyre2/), monomer in T conformation (blue) is shown. **b.** Superimposition of St_AcrD homology model to monomer in T conformation (turquoise) from Ec_AcrB crystal structure (PDB:4DX5). **c.** Location of putative substrate binding pockets of St_AcrD (sphere representation), access pocket (AP) in red; deep binding pocket (DBP) in orange, and the fusidic acid binding site in transmembrane helices 1 and 2 (TM1/TM2 region) in green. Figures were prepared with Pymol (https://pymol.org/).

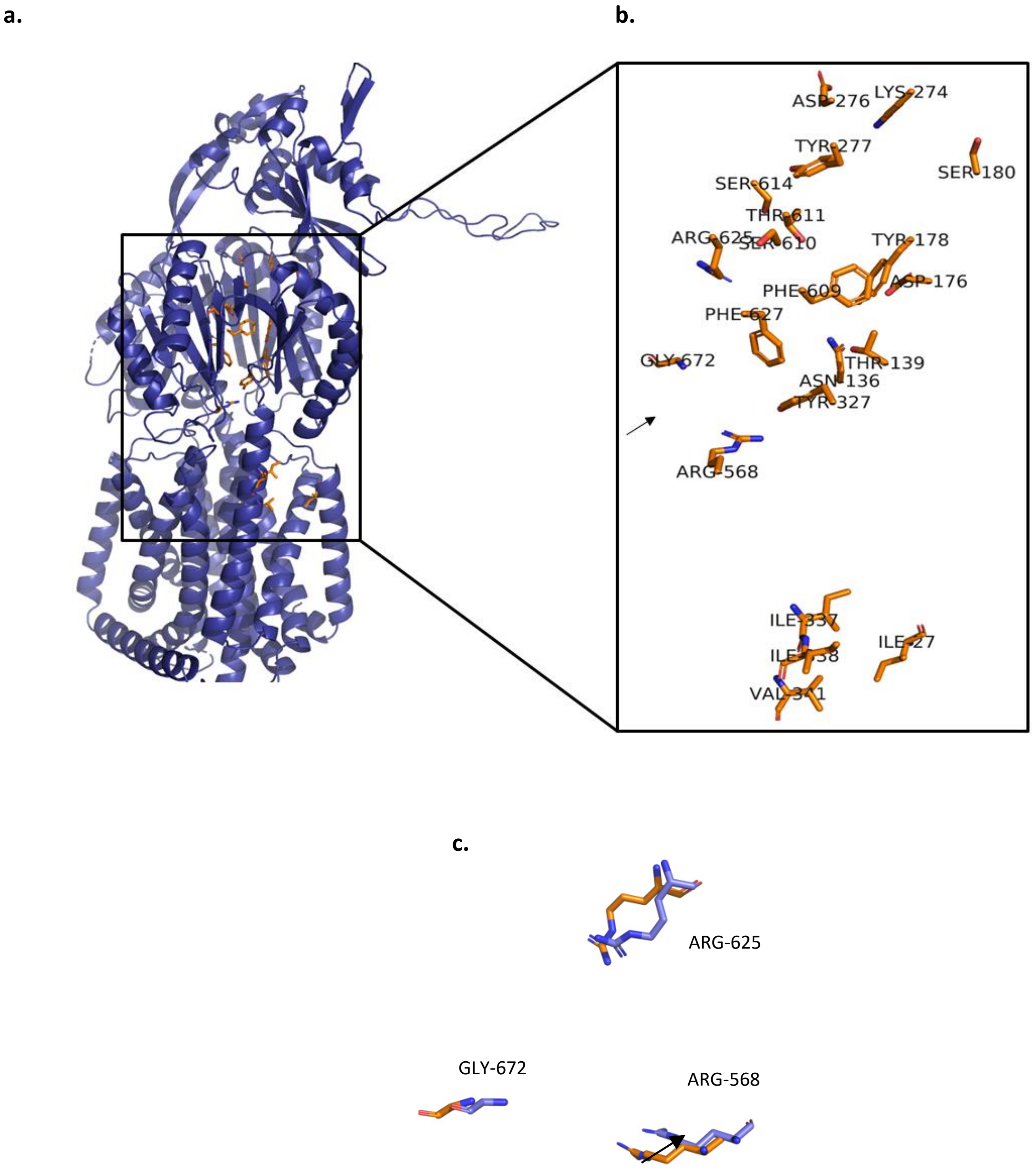

Figure S2. Putative substrate translocation pathway in St_AcrD and localization of the selected side chains for substitution. a. Homology model of St_AcrD in T conformation localizing putative residues (orange sticks) involved in substrate transport. **b.** Zoom in of target residues in the translocation pathway of St_AcrD. The black arrow indicated the entrance to the DBP from the AP **c.** Residue orientation of R568, R625 and G672 in the access pocket indicated in L (blue) and T (orange) conformations, respectively.

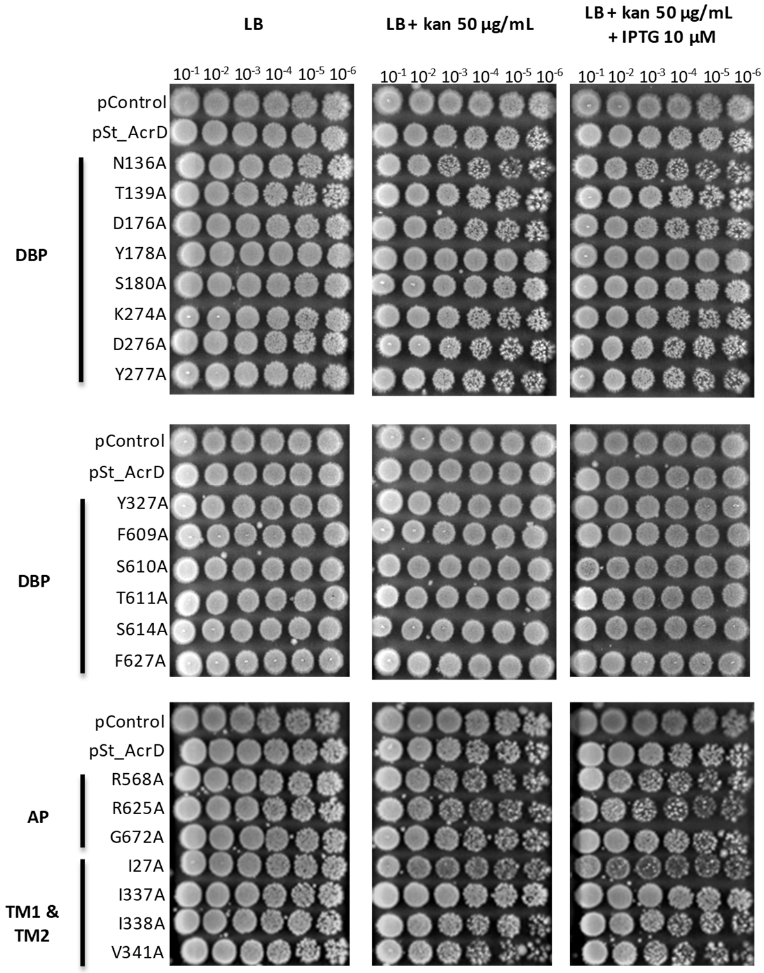

Figure S3. Plate dilution assay with *E. coli* BW25113 (DE3) Δ*acrB*Δ*acrD* harboring St_AcrD WT or the indicated Ala-substitution variants. Growth control of *E. coli* BW25113 (DE3) Δ*acrB*Δ*acrD* cells harboring St_AcrD wild type (pSt_AcrD) or Ala-variants on LB agar plates, and LB agar plates supplemented with 50 µg/mL kanamycin (kan) and 10 µM IPTG. pControl: empty vector (negative control); DBP: deep binding pocket; AP: access pocket; TM1/TM2 region: fusidic acid binding site. The images were recorded with the ImageQuant LAS4000 Imager. Subsequently the raw images were prepared using ImageJ.

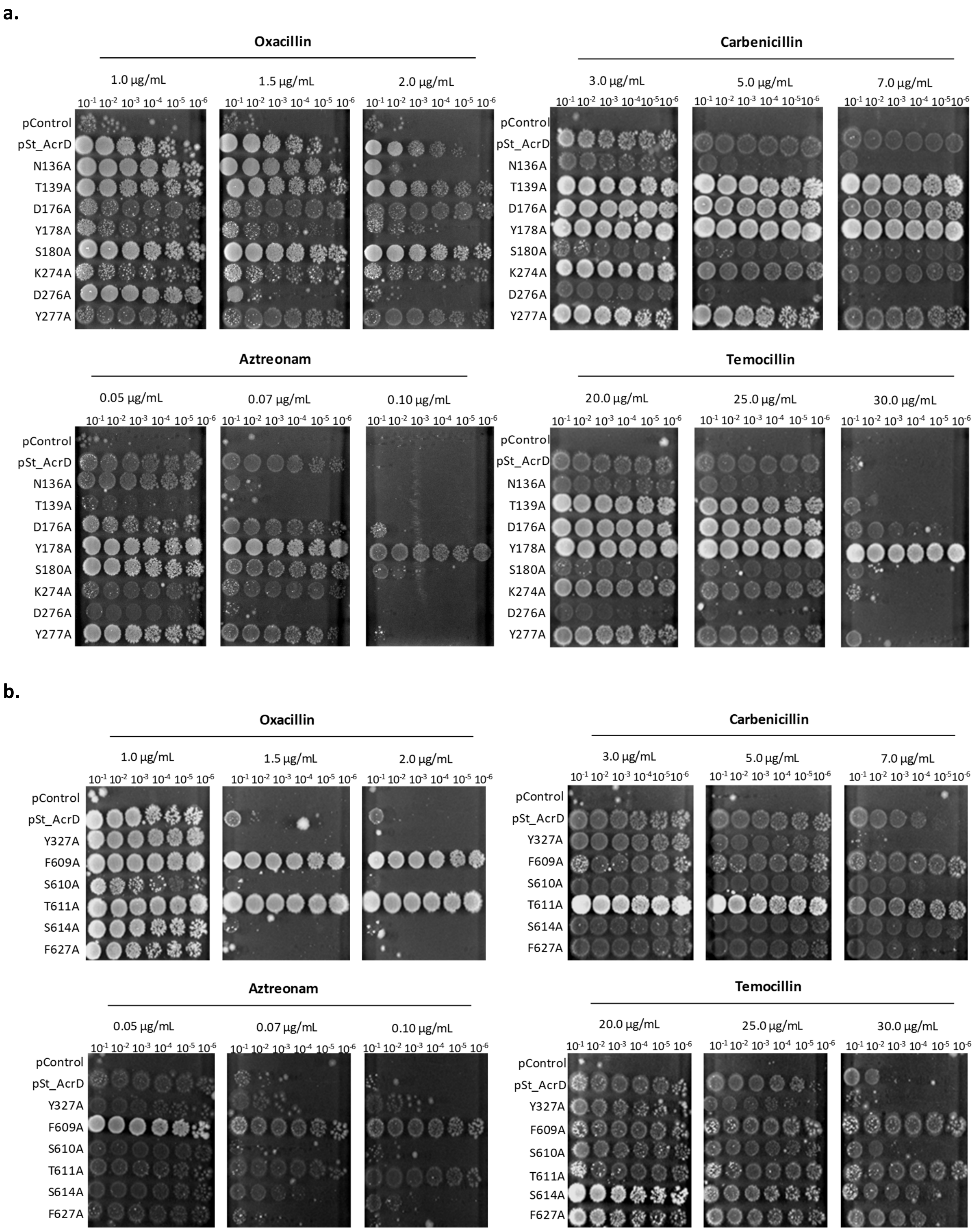

a.
Oxacillin
1.0 µg/mL
1.5 µg/mL
2.0 µg/mL
Carbenicillin
3.0 µg/mL
5.0 µg/mL
7.0 µg/mL
10-1 10-2 10-3 10-4 10-5 10-6
pControl
pSt_AcrD
N136A
T139A
D176A
Y178A
S180A
K274A
D276A
Y277A
Aztreonam
0.05 µg/mL
0.07 µg/mL
0.10 µg/mL
Temocillin
20.0 µg/mL
25.0 µg/mL
30.0 µg/mL
b.
Oxacillin
1.0 µg/mL
1.5 µg/mL
2.0 µg/mL
Carbenicillin
3.0 µg/mL
5.0 µg/mL
7.0 µg/mL
pControl
pSt_AcrD
Y327A
F609A
S610A
T611A
S614A
F627A
Aztreonam
0.05 µg/mL
0.07 µg/mL
0.10 µg/mL
Temocillin
20.0 µg/mL
25.0 µg/mL
30.0 µg/mL

.

c.

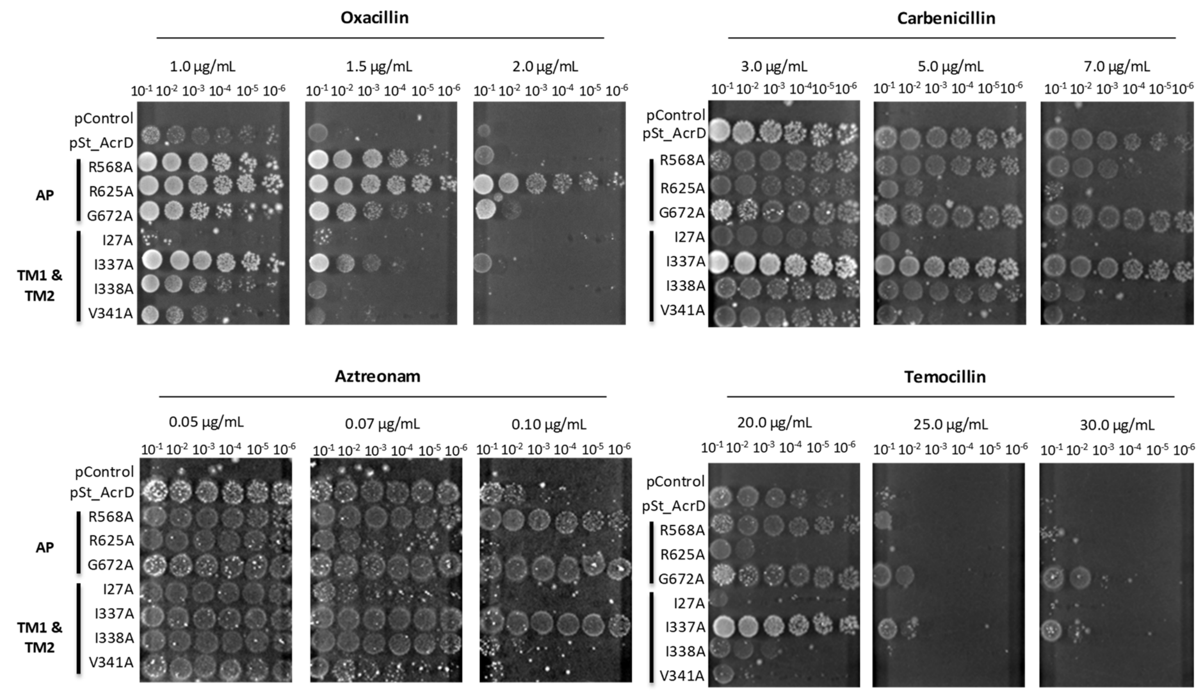

Figure S4. Resistance profiles against ß-lactams for *E. coli* BW25113 (DE3) Δ*acrB*Δ*acrD* harboring St_AcrD Ala-substitutions in the putative DBP, AP and in the TM1/TM2 region. Plate dilution assay of *E. coli* BW25113 (DE3) Δ*acrB*Δ*acrD* cells harboring St_AcrD wild type (pSt_AcrD) or deep binding pocket (DBP) Ala-variants from N136A to Y277A (**a.**), Y327A to F627A (**b.**) and (**c.**) Ala variant inside the access pocket (AP) and the TM1/TM2 region. All cells were grown on LB agar plates supplemented with 50 µg/mL kanamycin, 10 µM IPTG and the indicated antibiotic. pControl: empty vector (negative control). The images were recorded with the ImageQuant LAS4000 Imager. Subsequently the raw images were prepared using ImageJ.

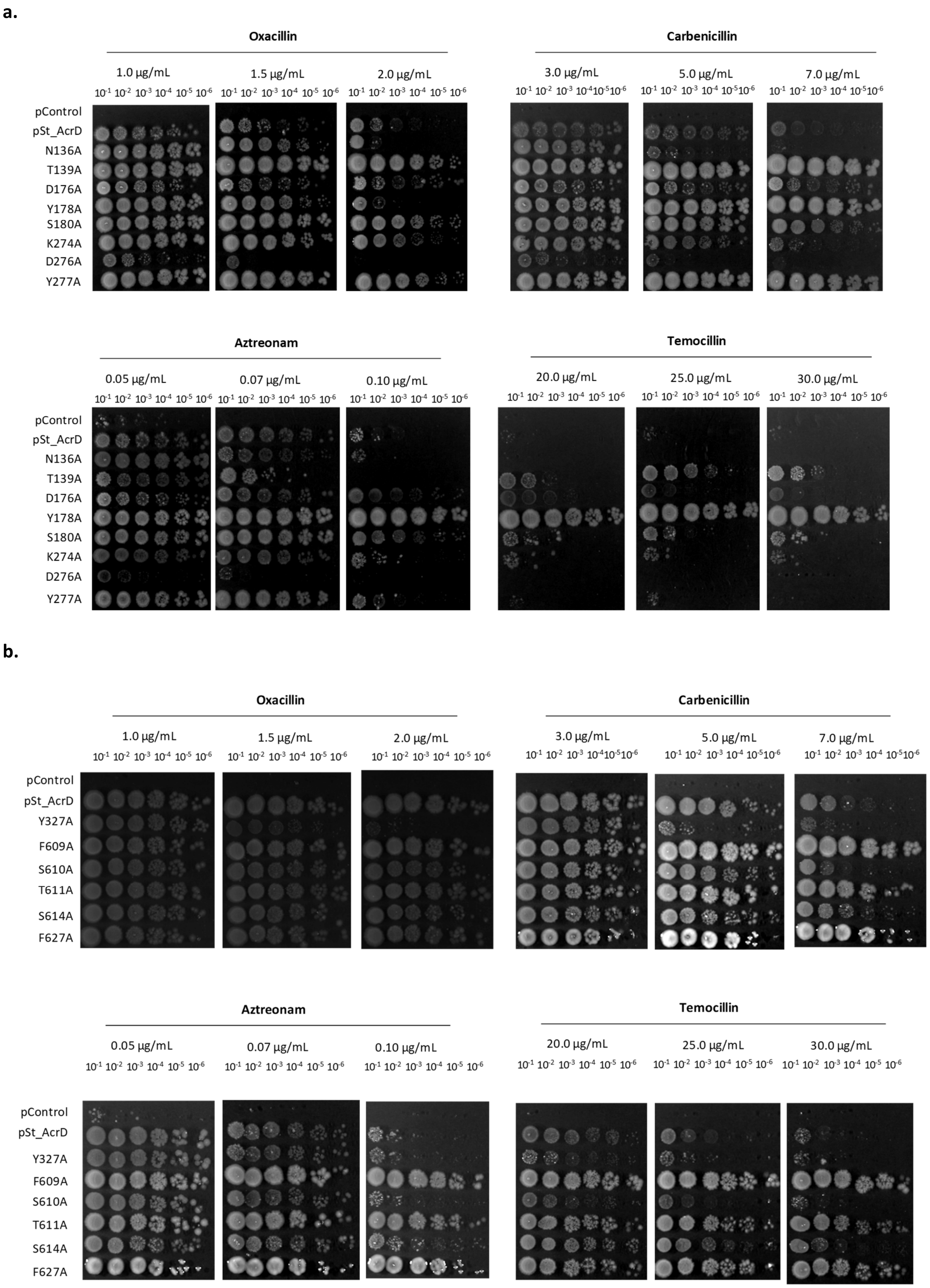
a.
Oxacillin
1.0 µg/mL
1.5 µg/mL
2.0 µg/mL
Carbenicillin
3.0 µg/mL
5.0 µg/mL
7.0 µg/mL
10-1 10-2 10-3 10-4 10-5 10-6
pControl
pSt_AcrD
N136A
T139A
D176A
Y178A
S180A
K274A
D276A
Y277A
Aztreonam
0.05 µg/mL
0.07 µg/mL
0.10 µg/mL
Temocillin
20.0 µg/mL
25.0 µg/mL
30.0 µg/mL
b.
Oxacillin
1.0 µg/mL
1.5 µg/mL
2.0 µg/mL
Carbenicillin
3.0 µg/mL
5.0 µg/mL
7.0 µg/mL
pControl
pSt_AcrD
Y327A
F609A
S610A
T611A
S614A
F627A
Aztreonam
0.05 µg/mL
0.07 µg/mL
0.10 µg/mL
Temocillin
20.0 µg/mL
25.0 µg/mL
30.0 µg/mL

c.

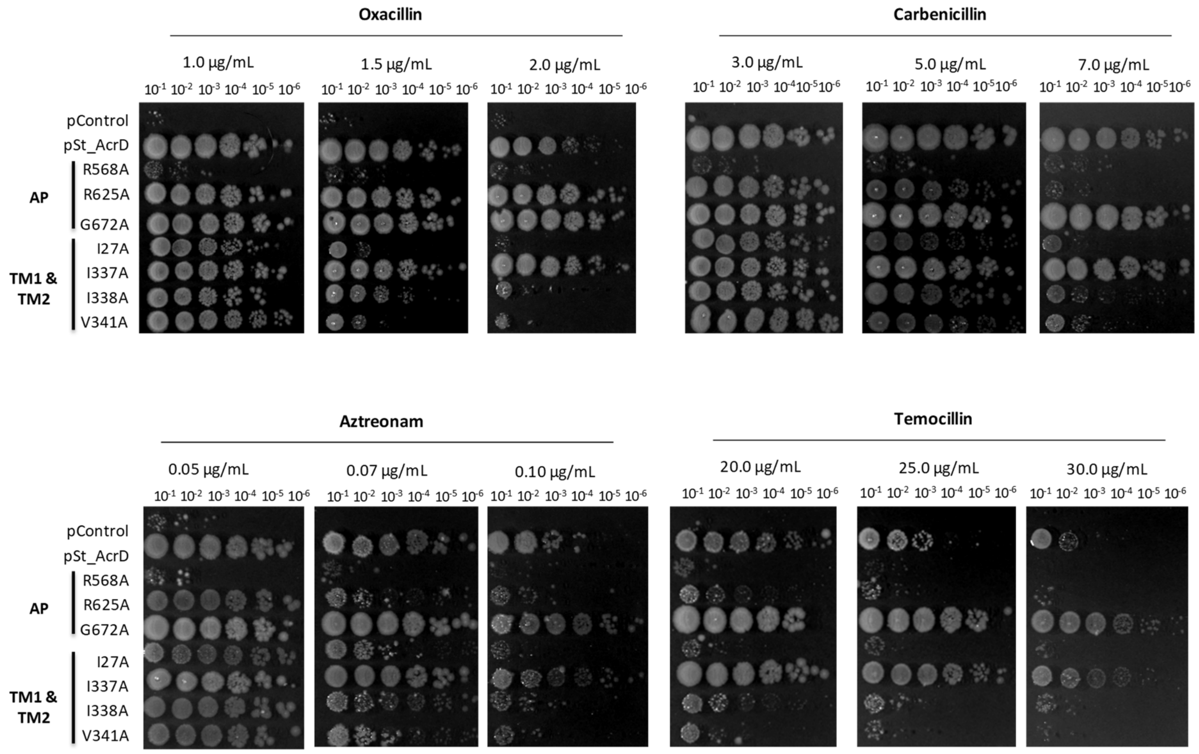

Figure S5. Resistance profiles against ß-lactams for *E. coli* BW25113 (DE3) Δ*acrB*Δ*acrD* harboring St_AcrD Ala-substitutions in the putative DBP (a. and b.) and AP and in TM1/TM2 region (c.). Plate dilution assay of *E. coli* BW25113 (DE3) Δ*acrB*Δ*acrD* cells harboring St_AcrD wild type (pSt_AcrD) or Ala-variants. All cells were grown on LB agar plates supplemented with 10 µM IPTG and the indicated antibiotic. pControl empty vector (negative control); AP: access pocket; TM1/TM2 region: fusidic acid binding site. The images were recorded with the ImageQuant LAS4000 Imager. In the experiment shown here the LB agar plates did not contain kanamycin as additional selective (in contrast to the experiments shown in Figure S4).

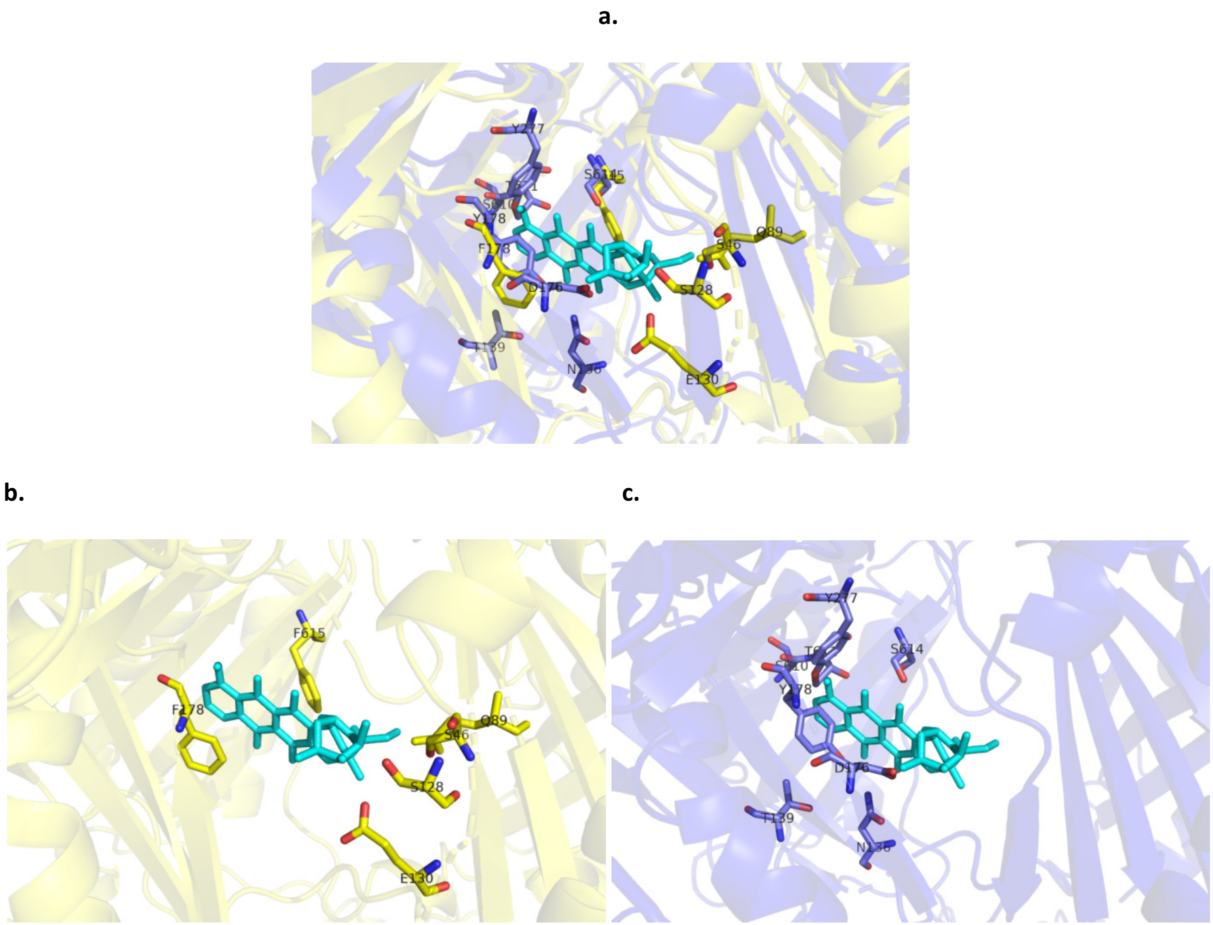

Figure S6. Comparison of DBP of Ec_AcrB (PDB: 4DX7) and St_AcrD (homology model). a. DBP superimposition of Ec_AcrB (yellow) and St_AcrD (blue) in the T conformation in the presence of doxorubicin (cyan). **b.** Ec_AcrB residues in the DBP involved in doxorubicin interaction. **c**. St_AcrD residues in the DBP provide a polar environment.

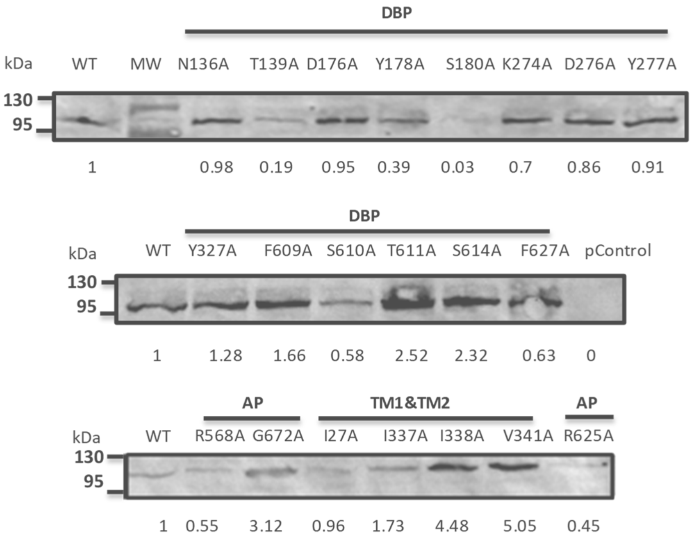

Figure S7. Production of St_AcrD WT and Ala-variants in *E. coli* BW25113 (DE3) Δ*acrB*Δ*acrD*. Anti-His Western blot analysis of whole cells harboring pControl (empty vector), St_AcrD WT or Ala-mutants with substitutions in the DBP, AP and TM1/TM2 region. Cells were resuspended from positive control LB agar plates supplemented with 50 µg/mL kanamycin and 10 µM IPTG. Samples were normalized to OD_{600} = 1 and 30 µL were loaded in each well of 10% SDS-PAA gels. Molecular weight marker (MW) = PageRuler Prestained Protein Ladder. The images were recorded with the ImageQuant LAS4000 Imager. Band intensity was quantified using the software ImageJ, and normalized to the wild-type St_AcrD (signal = 1) for every individual nitrocellulose membrane. Intensity numbers of the signal (bands) from the substitution variants is reported as a fraction from the St_AcrD WT signal. Brightness of the image shown here was increased by 30% compared to the original image.

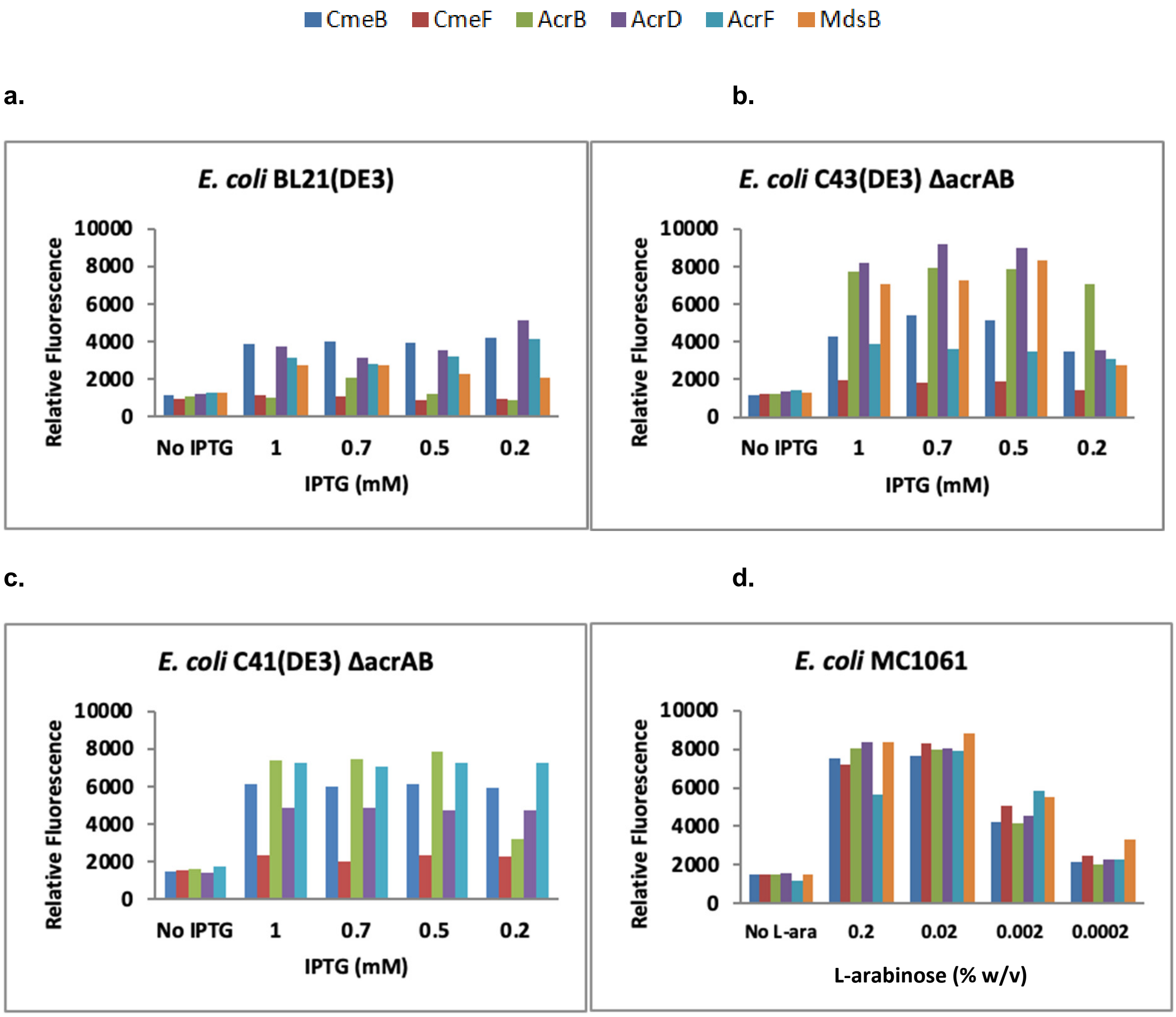

Figure S8. ***E. coli*** **strains and inducer concentration screening to produce RND-GFP fusion proteins.** **a.** *E. coli* BL21(DE3) **b.** *E. coli* C43(DE3)Δ*acrAB* **c.** *E. coli* C41(DE3)Δ*acrAB* and **d.** *E. coli* MC1061. Cells in experiments a. b. and c. were transformed with the p7XC3GH constructs and induced with IPTG. Cells in experiment d. were transformed with the pBXC3GH constructs and induced with L-arabinose. Whole-cell fluorescence measurements were done after 4 h post-induction and shown as Relative Fluorescence (Fluorescence arbitrary units/OD_{600}).

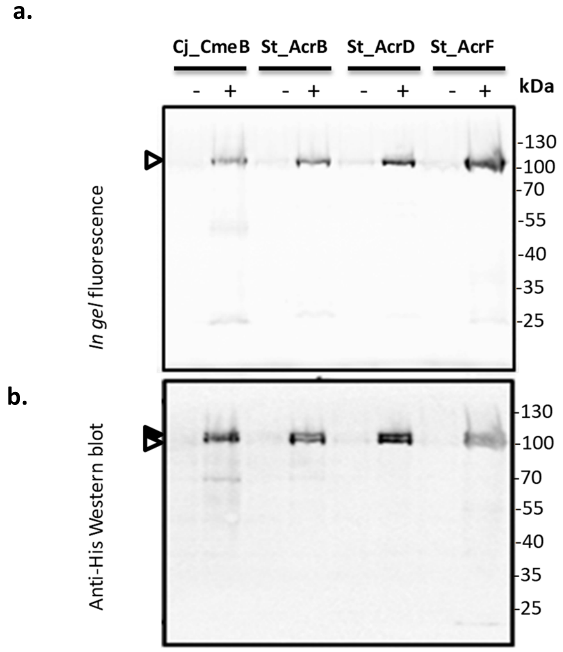

Figure S9. ***In gel fluorescence*** **and anti-His Western blot detection of RND-GFP fusion proteins produced in *E. coli*.** Whole cells expressing the fusion proteins according to the combinations described in Table S4, were collected and their cell density normalized to OD_{600} = 1. 10 µL of induced (+) and non-induced (-) samples were subjected to SDS-PAGE analysis using 10% polyacrylamide gels. **a.** *In gel fluorescence* and **b.** anti-His Western blot analysis were performed on the same gel. Arrows indicate the fluorescent and therefore correctly folded species (white) and misfolded protein (black). Molecular weight marker (MW)= PageRuler Prestained Protein Ladder (Thermo Scientific). The figures were produced using the ImageQuant LAS4000 Imager (excitation: 460 nm, emission: 510 nm).

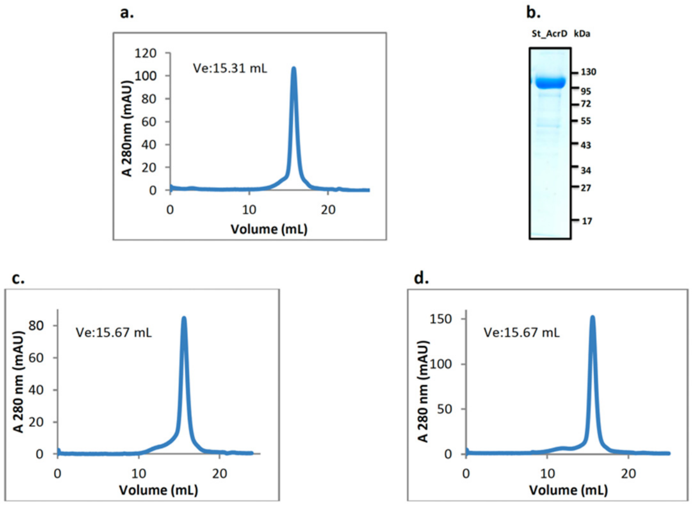

Figure S10. Purification of St_AcrD. SEC profiles of freshly purified protein (**a.**) and upon storage at 4°C and 17°C for one week (**c.** and **d.**, respectively). Protein (0.30-0.35 mg) was applied to a Superose 6 15/300 increase column equilibrated in 20 mM Tris-Cl, pH 7.5, 150 mM NaCl, 0.04 % w/v DDM buffer with a flow rate of 0.6 mL/min. Ve=elution volume. **b.** Coomassie-stained 10% SDS-PAA gel of purified St_AcrD (13 µg). Molecular weight marker (kDa)= PageRuler Prestained Protein Ladder (ThermoScientific).

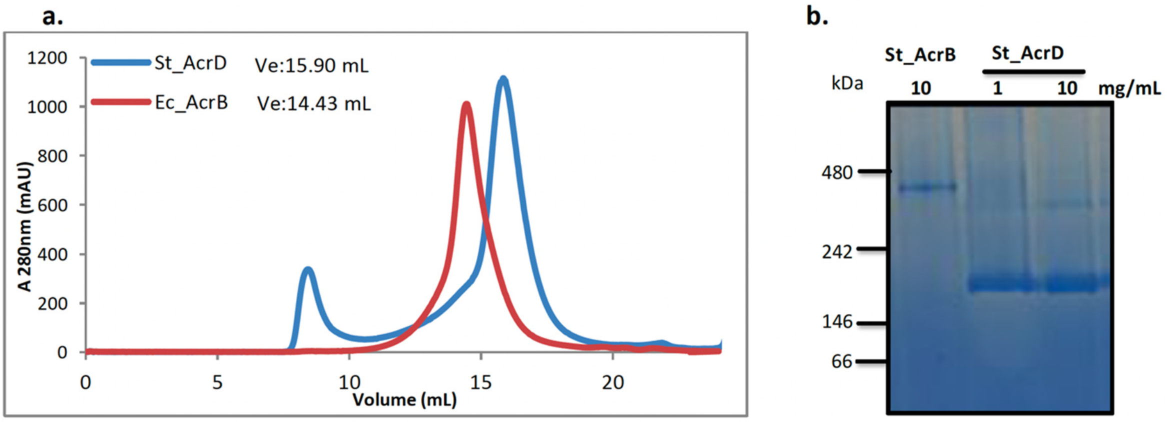

Figure S11. Oligomeric state of St_AcrD. a. SEC profiles of purified St_AcrD and Ec_AcrB (4 mg). Superose 6 15/300 increase column was equilibrated in 20 mM Tris-Cl, pH 7.5, 150 mM NaCl, 0.04 % w/v DDM buffer with a flow rate of 0.6 mL/min. Ve=elution volume. **b.** Purified St_AcrB (5µg) and St_AcrD (10 µg) were loaded on 3-12% BisTris Blue NativePAGE gels (ThermoFisher). Molecular weight marker = NativeMark Unstained Protein Ladder (ThermoFisher).
