# Supplementary material for: Characterization and Molecular Determinants for β-Lactam Specificity of the Multidrug Efflux Pump AcrD from Salmonella typhimurium
Source: Antibiotics (Basel). 2021 Dec 6;10(12):1494. doi: 10.3390/antibiotics10121494 (PMC8699017; doi:10.3390/antibiotics10121494)
Supplement: Supplementary file 1 [file antibiotics-10-01494-s001.zip › Supplementary_Tables_2021_11_12.pdf]

Table S1. Comparison of identities and similarities of RND multidrug transporters of *E. coli*, *C. jejuni* and *S. typhimurium*. Identity and similarity percentage (%I and %S) were obtained from protein sequence alignments employing Protein Blast server (https://blast.ncbi.nlm.nih.gov/Blast.cgi). Closely related homologues are highlighted in green.

RND transporter	*E. coli*									
	AcrB		AcrD		AcrF		MdtB		MdtC	
	%I	%S	%I	%S	%I	%S	%I	%S	%I	%S
Campylobacter jejuni										
Cj_CmeB	42	63	43	63	42	61	28	48	30	51
Cj_CmeF	26	48	26	48	27	48	28	48	27	49
Salmonella typhimurium										
St_AcrB	95	97	65	80	79	89	29	49	29	50
St_AcrD	66	80	94	97	63	77	29	50	29	49
St_AcrF	81	91	64	78	89	94	29	50	28	49
St_MdsB	41	64	40	62	42	63	30	51	31	50
St_MdtB	28	47	29	50	28	49	91	96	49	67
St_MdtC	28	49	28	49	29	50	49	67	92	96

Table S2. Primers used for FX-cloning and site-directed mutagenesis
All primers were obtained from *Microsynth AG*.

Primers used for FX-cloning

Primer	Sequence (5´→ 3´)
Fw cmeB	ATATATGCTCTTCTAGTTTTCTAAATTTTTTATCGA
Rev cmeB	TATATAGCTCTTCATGCTTCATGAACCTTACCTCTTT
Fw cmeF	ATATATGCTCTTCTAGTTTTAAACTAGCTATAAACCG
Rev cmeF	TATATAGCTCTTCATGCATTTAATTTTTCTCTCTCAT
Fw acrB	ATATATGCTCTTCTAGTCCTAATTTCTTTATCGATCG
Rev acrB	TATATAGCTCTTCATGCGCGATGTTCTGTCGAATGAC
Fw acrD	ATATATGCTCTTCTAGTGCGAATTTTTTTATCGATCG
Rev acrD	TATATAGCTCTTCATGCTTTCGGGCGCGGCTTCAGCG
Fw acrF	ATATATGCTCTTCTAGTGCAAACTTTTTTATTAGACG
Rev acrF	TATATAGCTCTTCATGCATCATGATGGCGATTAAATC
Fw mdsB	ATATATGCTCTTCTAGTAAATTCACCCACTTTTTCAT
Rev mdsB	TATATAGCTCTTCATGCTGCTTGCTGATCATGCGAAT
Dfw1AcrD	ATATATGCTCTTCTAGTGCGAATTTTTTTATCGATCG
Drev1 AcrD	TATATAGCTCTTCATGCATTATTAATGATGATGATGA
Dfw 2AcrD	ATATATGCTCTTCTAGTCACCATCATCATCACCATCATC
Drev2 AcrD	TATATAGCTCTTCATGCTTATTATTCGTGCCATTCG

Primers used for site-directed mutagenesis

Primer	Sequence (5´→ 3´)
N136A-Fw	GCTATTTTGACCATCGCTTT
N136A-Rev	GGTATCGCCCGTTTTGC
T139A-Fw	GCCATCGCTTTCGTCTCT
T139A-Rev	CAAAATATTGGTATCGCCCGT
D176A-Fw	GCCGCTTATGGTTCACAGT
D176A-Rev	AATATCGCCGACGCCGT
Y178A-Fw	GCTGGTTCACAGTACTCTA
Y178A-Rev	AGCGTCAATATCGCCGA
S180A-Fw	GCACAGTACTCTATGCGT
S180A-Rev	ACCATAAGCGTCAATATCG
K274A-Fw	CGTATGACTACCTCAGCCG
K274A-Rev	CTTCCGCCCCCAGCTC
D276A-Fw	GCCTACCTCAGCCGTTT
D276A-Rev	ATACTTTTCCGCCCCCA
Y277A-Fw	GCCCTCAGCCGTTTTAACG
Y277A-Rev	GTCATACTTTTCCGCCCCC
Y327A-Fw	GCTGAAACCACCTCCTTTG
Y327A-Rev	CGCGATCTTGTATTCCAGG
F609A-Fw	CCTCGACGGTAGGTTCCG
F609A-Rev	CGACCGACATAATGTTGTCTT
S610A-Fw	GCGACGGTAGGTTCCGG
S610A-Rev	GAAGACCGACATAATGTTGTCT
T611A-Fw	CGGTAGGTTCCGGCCCT
T611A-Rev	CCGAGAAGACCGACATAATGT
S614A-Fw	GGCCCTGGCGGGAATG
S614A-Rev	GGCACCTACCGTCGAGAAGA
P616A-Fw	GCTGGCGGGAATGGGCAA
P616A-Rev	GCCGGAACCTACCGTCG
F627A-Fw	GCTGTTCGCTTGAAAGACT
F627A-Rev	CATGCGCGCGACGTTTT
R568A-Fw	GCGGGCATGTTCACTACGT
R568A-Rev	ATCTTCCTGCGGCAGAAA
R625A-Fw	GCCATGTTTGTTCGCTTGA
R625A-Rev	CGCGACGTTTTGCCCAT
G672A-Fw	CTCTGGGCAGCTCCGCCG
G672A-Rev	CGCTAATTGCCGGCGGGC
I27A-Fw	GCTTTCTCTTTACCTGTTGAACA
I27A-Rev	GGCTAACGCCCCTGTCA
I337A-Fw	GCTATCGATGTGGTCAAAA
I337A-Rev	CGAGGCTTTGACAAAGG
I338A-Fw	GCCGATGTGGTCAAAACGT
I338A-Rev	AATCGAGGCTTTGACAAAGG
V341A-Fw	GCCAAAACGTTGCTGGA
V341A-Rev	CACATCGATAATCGAGGCT
Fwmut_Y178A	GGCGATATTGACGCTGCTGGTTCACAGTACTCT
Revmut_Y178A	AGAGTACTGTGAACCAGCAGCGTCAATATCGCC

Table S3. Multidrug RND transporters from *Campylobacter jejuni* and *Salmonella typhimurium*. Number of amino acid residues and molecular weight of the proteins were calculated with ProtParam (https://web.expasy.org/protparam/). The KEGG identifier (https://www.genome.jp/kegg/) of each gene is given in brackets after the gene name.

Gene	Protein	Amino acid residues	Molecular weight (kDa)
Campylobacter jejuni			
cmeB (Cj0366c)	Cj_CmeB	1040	113
cmeF (Cj1033)	Cj_CmeF	1005	112
Salmonella typhimurium			
mdsB (STM0351)	St_MdsB	1055	114
acrB (STM0475)	St_AcrB	1049	114
mdtB (STM2127)	St_MdtB	1040	112
mdtC (STM2128)	St_MdtC	1026	111
acrD (STM2481)	St_AcrD	1037	113
acrF (STM3391)	St_AcrF	1037	112

Table S4. Cloning and expression constructs of RND transporters produced by FX-cloning. PCR products of the target genes were cloned initially in pINITcat and subsequently sub-cloned into the expression vectors p7XC3GH and pBXC3GH.

Cloning constructs	Expression constructs	
pINITcat-cmeB	p7XC3GH-cmeB<br>p7XC3H-cmeB	pBXC3GH-cmeB
pINITcat-cmeF	p7XC3GH-cmeF	pBXC3GH-cmeF
pINITcat-acrB	p7XC3GH-acrB<br>p7XC3H-acrB	pBXC3GH-acrB
pINITcat-acrD	p7XC3GH-acrD<br>p7XC3H-acrD	pBXC3GH-acrD
pINITcat-acrF	p7XC3GH-acrF	pBXC3GH-acrF
pINITcat-mdsB	p7XC3GH-mdsB	pBXC3GH-mdsB

Table S5. Summary of optimized conditions to produce RND-GFP fusion proteins in *E. coli*.

RND	*E. coli* strain	Inducer	Time	Temp.	Medium
Cj_CmeB	C41(DE3)Δ*acrAB*	0.5 mM IPTG	16 h	25°C	TB
Cj_CmeF	MC1061	0.02% L-arabinose			
St_AcrB	C43(DE3)Δ*acrAB*	0.5 mM IPTG			
St_AcrD	C43(DE3)Δ*acrAB*	0.5 mM IPTG			
St_AcrF	C41(DE3)Δ*acrAB*	0.5 mM IPTG			
St_MdsB	MC1061	0.02% L-arabinose			
